# Pulse frequency dependency of photobiomodulation on the bioenergetic functions of human dental pulp stem cells

**DOI:** 10.1038/s41598-017-15754-2

**Published:** 2017-11-21

**Authors:** Hong Bae Kim, Ku Youn Baik, Pill-Hoon Choung, Jong Hoon Chung

**Affiliations:** 1 Department of Biosystems & Biomaterials Science and Engineering, Seoul National University, Seoul, 08826 Republic of Korea; 2 Electrical and Biological Physics, Kwangwoon University, Seoul, 01897 Republic of Korea; 3 Department of Oral and Maxillofacial Surgery and Dental Research Institute, School of Dentistry, Seoul National University, Seoul, 03080 Republic of Korea; 4 Research Institute for Agriculture and Life Sciences, Seoul National University, Seoul, 08826 Republic of Korea

## Abstract

Photobiomodulation (PBM) therapy contributes to pain relief, wound healing, and tissue regeneration. The pulsed wave (PW) mode has been reported to be more effective than the continuous wave (CW) mode when applying PBM to many biological systems. However, the reason for the higher effectiveness of PW-PBM is poorly understood. Herein, we suggest using delayed luminescence (DL) as a reporter of mitochondrial activity after PBM treatment. DL originates mainly from mitochondrial electron transport chain systems, which produce reactive oxygen species (ROS) and adenosine triphosphate (ATP). The decay time of DL depends on the pulse frequencies of applied light, which correlate with the biological responses of human dental pulp stem cells (hDPSCs). Using a low-power light whose wavelength is 810 nm and energy density is 38 mJ/cm^2^, we find that a 300-Hz pulse frequency prolonged the DL pattern and enhanced alkaline phosphatase activity. In addition, we analyze mitochondrial morphological changes and their volume density and find evidence supporting mitochondrial physiological changes from PBM treatment. Our data suggest a new methodology for determining the effectiveness of PBM and the specific pulse frequency dependency of PBM in the differentiation of hDPSCs.

## Introduction

Photobiomodulation (PBM) therapy generally uses light in the red or near-infrared (NIR) region, with wavelengths ranging from 600 to 700 nm and from 780 to 1,100 nm, respectively. The output power varies widely from 1 to 500 mW^1^. PBM has been shown to influence a wide variety of cellular functions, including gene expression, growth and proliferation, survival, and differentiation^2–6^. These functions are primarily mediated by raising the levels of adenosine triphosphate (ATP), which increases the mitochondrial membrane potential, cyclic adenosine monophosphate, calcium (Ca^2+^), and reactive oxygen species (ROS) and activates transcription factors^7,8^. Cytochrome c oxidase appears to be the primary photoacceptor and transducer of photosignals in these regions of the light spectrum^9^. The accepted light energy activates the cytochrome c oxidase and triggers a series of biochemical cascades that improve cellular functions^10^.

PBM can be classified into two modes by its continuity: continuous wave (CW) and pulsed wave light (PW). Most previous studies have used CW-PBM to aggressively promote the proliferation and differentiation of stem cells^4,6,11–24^, beginning with dental treatment^21^. CW-PBM typically uses low power density, from 5 mW/cm^2^ to 5 W/cm^2^ ^25^, to prevent thermal effects in intracellular molecules. However, PW-PBM is more effective in maintaining an a-thermal environment due to the quenching periods, that is, OFF times. PW-PBM also enables the light to penetrate more deeply into a biological system than CW-PBM because it uses higher peak power while keeping the total energy the same^26^. In addition, PW-PBM can promote light–biological system interactions. Some fundamental frequencies in biological systems, in the range of tens to hundreds Hz, are similar to the pulsing frequencies used in PW-PBM^26^.

On the other side, the responsiveness of biological systems to PBM can be identified using delayed luminescence (DL), which is measured in the form of optical photons emitted after the illumination source is switched off. Thus, DL is a spectral emission from the optical range to NIR, and its intensity is various orders of magnitude^27^. DL demonstrates cellular reduction/oxidation (redox) states in relation to cytochrome c oxidase, which produces ROS in the mitochondrial respiratory chain^28,29^. Because the cellular redox state appears to differ in the proliferation and differentiation phases of a cell, DL can be used to determine cellular phases^30–32^. The cellular phase is associated with further transient increases in cellular ROS production, which also affects DL^33^.

**Figure 1:**
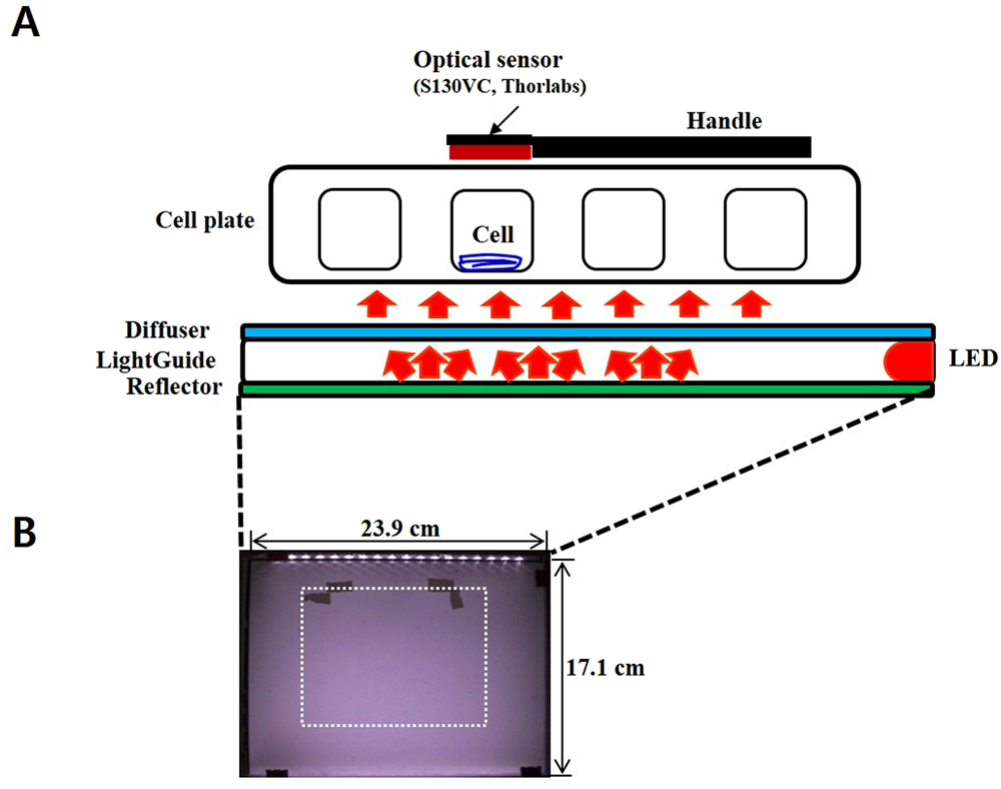
(**A**) Schematics of PBM device and DL detection system. The light from an LED traveled via a reflector, a light-guide, and a diffuser into cells in PBS. DL was measured on top of the cell culture plate during and after PBM. (**B**) A photograph of the PBM system with the homogenous center area marked with white dotted lines.

In this study, we used DL to monitor the responses of stem cells to an in-house-fabricated laser device (Fig. 1) with a light wavelength of 810 nm and an energy density of 38 mJ/cm^2^. The physiological states of hDPSCs were assessed after PBM treatment and compared with DL signals. PW-PBM induced more significant changes in the differentiation of hDPSCs and created longer-lasting DL from the cells than CW-PBM. In addition, specific pulse frequency dependencies appeared. These results suggest that the pattern of PBM, in addition to the light intensity, could be important for biological applications.

## Results

### PBM-mediated ROS production, proliferation, and alkaline phosphatase activity

**Figure 2:**
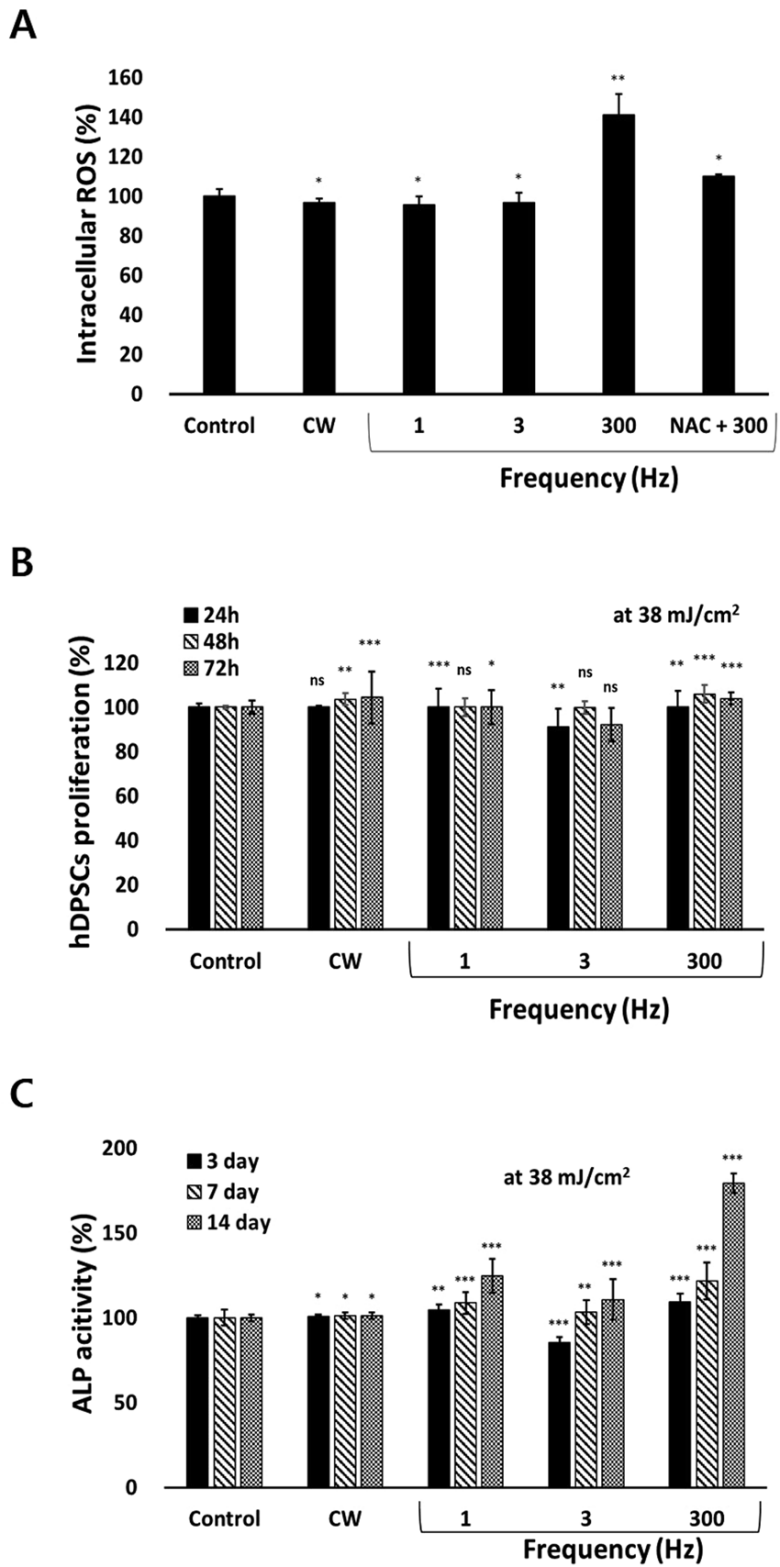
Biological responses of hDPSCs to CW- and PW-PBM with different frequencies. (**A**) Intracellular ROS was measured with H2DCFDA. NAC was used to confirm the fluorescence signals. (**B**) Proliferation of hDPSCs was assessed with WST-1 at the elapsed times of 1, 2, and 3 days. (**C**) Alkaline phosphatase activity was measured at the elapsed times of 3, 7, and 14 days. Statistical significance is marked with *, **, and *** when *P* < 0.05, *P* < 0.01, and *P* < 0.001, respectively.

To associate the cellular redox state with PBM, PBM-mediated ROS production was assessed. Increased production of ROS was observed in hDPSCs after treatment with 300-Hz PW-PBM (*P* < 0.0095), whereas no changes were noted after CW-PBM (Fig. 2A). Incubation with a ROS scavenger, N-acetyl cysteine (NAC), reduced ROS detection after 300-Hz PW-PBM (Fig. 2A).

hDPSCs were subjected to CW- and PW-PBM treatment for 3 days to assess their proliferation. hDPSCs subjected to CW-PBM showed a slight increase in proliferation (*P* < 0.0009; Fig. 2B), whereas those that received PW-PBM showed either a slight increase or a decrease in the amount of proliferation (*P* < 0.0032 for 3-Hz PBM, *P* < 0.00012 for 300-Hz PBM; Fig. 2B). The proliferation of hDPSCs did not reflect the level of PBM-mediated ROS.

The early differentiation of hDPSCs by PBM treatment of 38 mJ/cm^2^ was assessed using alkaline phosphatase (ALP) activity after 14 days of treatment. Significantly enhanced ALP activity was observed in the hDPSCs subjected to PW-PBM treatment (*P* < 0.001 for 1 Hz, *P* < 0.012 for 3 Hz, *P* < 0.001 for 300 Hz; Fig. 2C), whereas no alteration was observed from CW-PBM on 14 day after PBM treatment. Interestingly, the ALP activity of hDPSCs well reflected the result from 300-Hz PBM-mediated ROS production.

### Delayed luminescence

**Figure 3:**
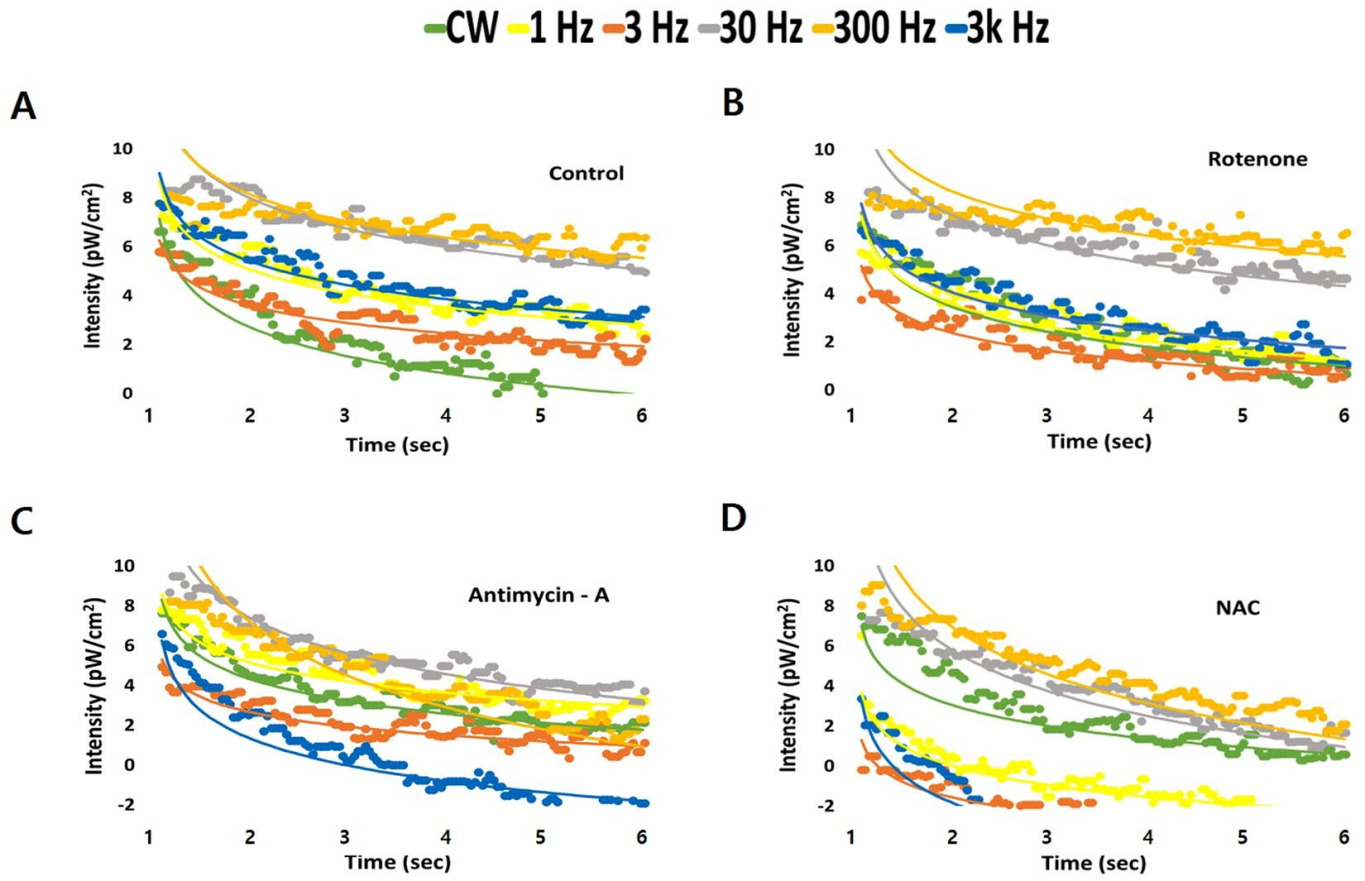
DL data from hDPSCs in response to PBM according to pulse frequency. The measured DL in the period between 1 s and 6 s after PBM was plotted with theoretical fittings. DL was measured when hDPSCs were in PBS with no treatment (**A**), with pre-treatment in rotenone (**B**), with pre-treatment in AMA (**C**), and with pre-treatment in NAC (**D**).

To confirm to the effects of the 810-nm wavelength PBM treatment on the transfer of energy and related chemical responses, we measured DL from the end of light irradiation. Figure 3A shows the patterns of DL in hDPSCs in Dulbecco’s phosphate-buffered saline (DPBS) for 5 seconds after CW-PBM or PW-PBM at frequencies of 1, 3, 30, 300, and 3000 Hz. Interestingly, all the frequencies of PW-PBM induced more lagged luminescence than CW-PBM, which showed that some chemical activations result from PW-PBM treatment. Though the total energy transferred to cells was the same, the energy per pulse decreased as the frequency increased. We found pulse frequency dependencies in the DL patterns; however they were not related to the total energy or energy per pulse. 30- and 300-Hz PBM treatment induced highly lagged DL, and 3-Hz PBM treatment had the least effect on DL.

Considering previous reports^27,34–39^ that showed a relationship between mitochondrial activity and DL, we used rotenone, which inhibits the transfer of electrons from iron-sulfur centers in mitochondrial complex I to ubiquinone, or antimycin-A (AMA), which inhibits the oxidation of ubiquinone in mitochondrial complex III. In both cases, time delays after CW-PBM treatment became slower, but those after PW-PBM treatments were faster than in cells untreated by chemicals (Fig. 3B,C). However, it is clear that the effect of AMA was more significant in reducing DL intensity than DL delays. The pulse frequency dependencies changed differently, indicating the presence of a response protein to PW-PBM. The order of the delay times was kept similar with the addition of rotenone but changed slightly with AMA. Because many reports have shown that PBM-induced biological responses are mediated by intracellular ROS^34,36,38,39^, we examined the effects of NAC on DL. Figure 3D shows that DL after CW-PBM treatment was almost the same with and without NAC, but delay times after PW-PBM became faster at all frequencies. Especially at 1, 3, and 3000 Hz, the delays dropped abruptly compared with the control levels.

**Figure 4:**
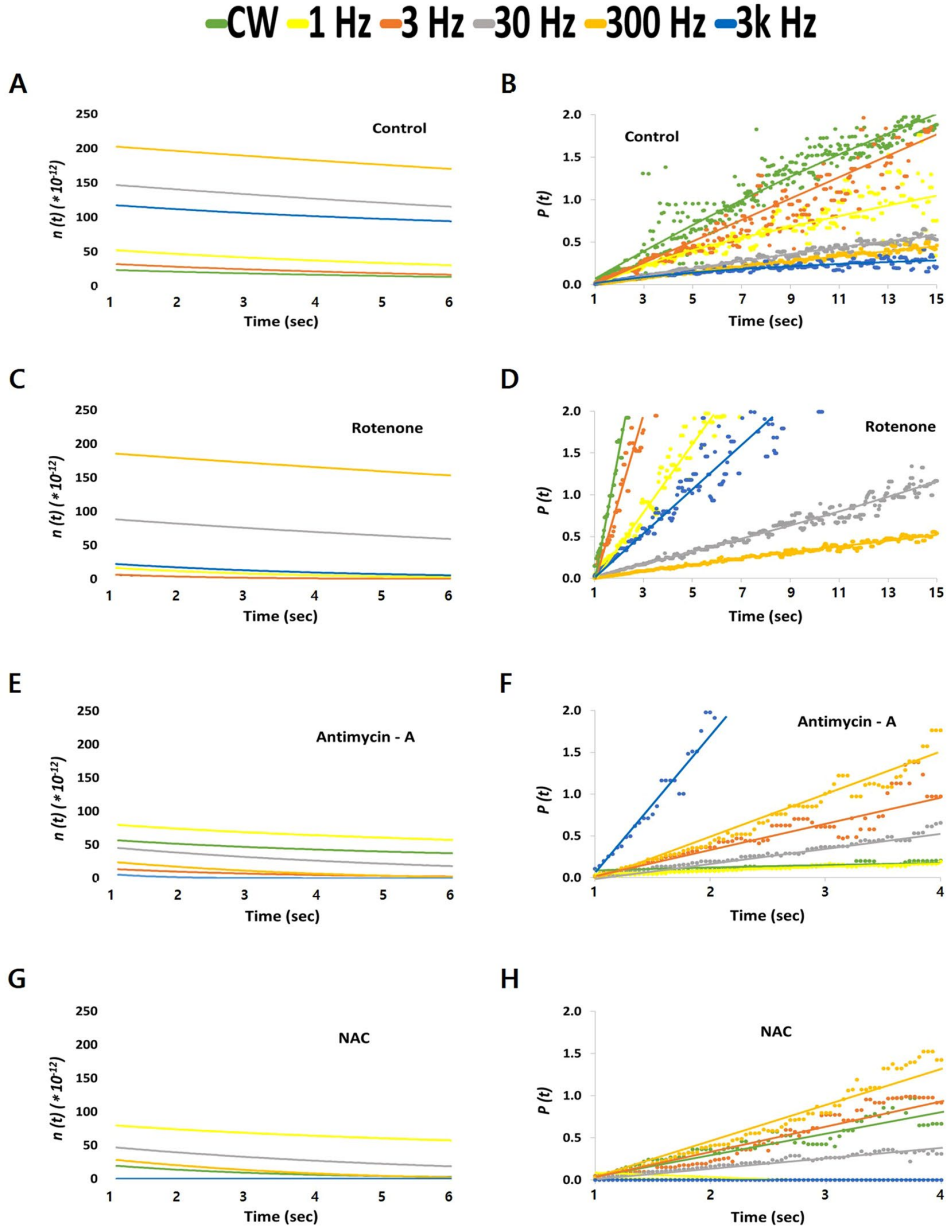
The temporal trend of the decay probability *P*(*t*) (**A**,**C**,**E**,**G**) and the degree of excitation *n*(*t*) (**B**,**D**,**F**,**H**) from the DL shown in Fig. 3. Data were analyzed from the DL measured when hDPSCs were in PBS with no treatment (**A**,**B**), with pre-treatment in rotenone (**C**,**D**), with pre-treatment in AMA (**E**,**F**), and with pre-treatment in NAC (**G**,**H**). The time scales of the *P*(*t*) were changed for clear discrimination.

To clearly understand the effects of PBM treatment on DL, we transformed the measured DL intensity, *I*(*t*), into the degree of the excitation level, *n*(*t*), and the decay probability *P*(*t*). The *n*(*t*) represents how many excited molecules (expected to emit DL) remain, and *P*(*t*) indicates the decrease rate in *n*(*t*) over time. The *n*(*t*) plots show clearly separated values between different frequencies whose *I*(*t*) curves were difficult to distinguish. Fig. 4A shows that the *n*(*t*) value was highest after 300-Hz PW-PBM and lowest after CW-PBM. The *P*(*t*) results were the reverse of the *n*(*t*) results; the value was highest after CW-PBM and lowest after 300-Hz PW-PBM (Fig. 4B). When hDPSCs were subjected to rotenone, the *n*(*t*) became lower and *P*(*t*) became higher; however, the order among the frequencies remained the same (Fig. 4C,D). When hDPSCs were subjected to AMA, the high *n*(*t*) values with 30- and 300-Hz PW-PBM became dramatically lower than those with other frequencies (Fig. 4E,F). A similar decrease was observed with NAC (Fig. 4G,H). On the other hand, the probabilities of the control hDPSCs were almost equally distributed with the pulse frequency of PW-PBM.

### Morphological changes in mitochondria from PBM treatment

**Figure 5:**
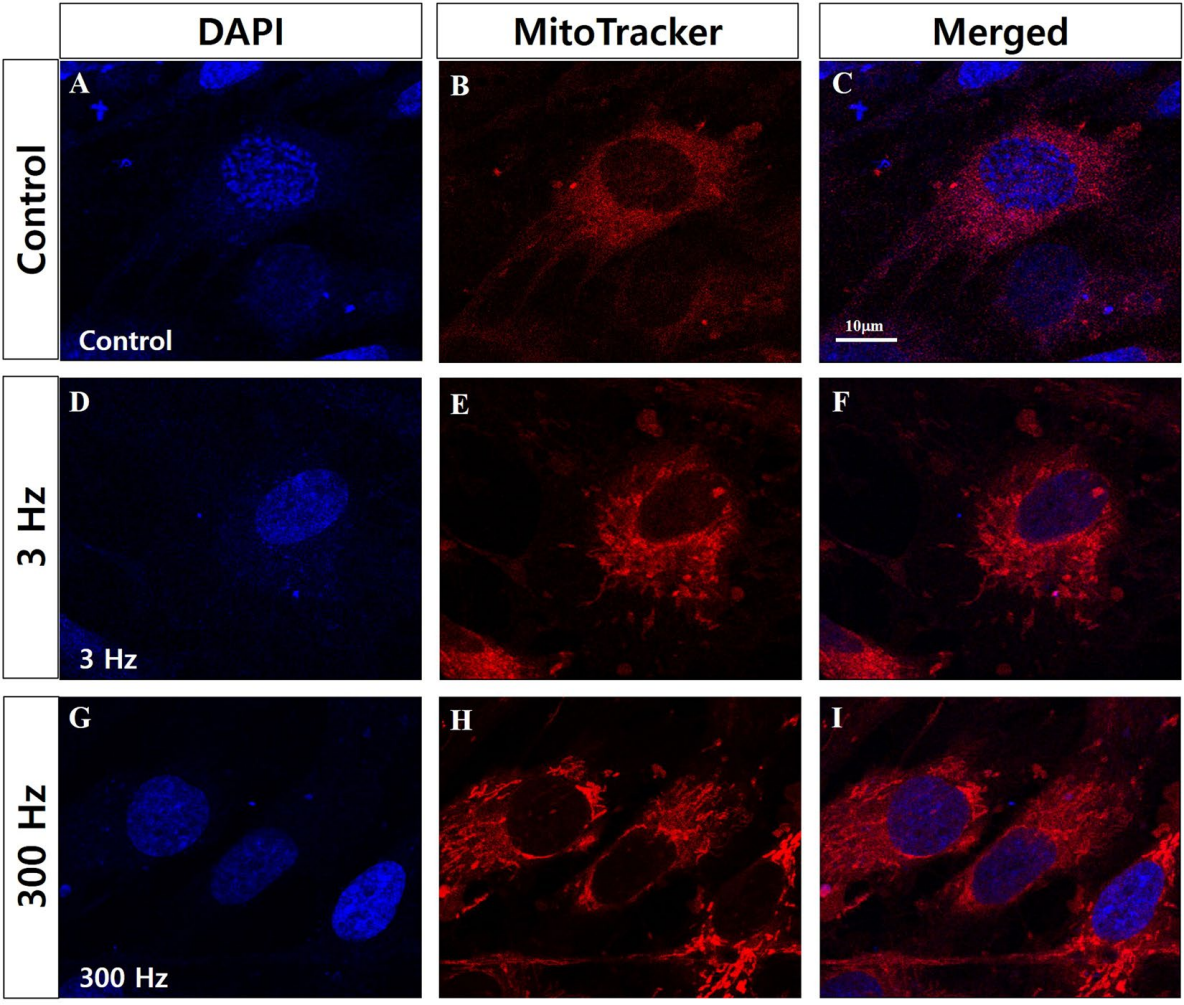
Confocal microscopic images of mitochondria in hDPSCs 3 days after PBM treatments. Nuclei were stained with 4′,6-diamidino-2-phenylindole (DAPI), and mitochondria were stained with MitoTracker Red. Control hDPSCs (**A–C**), hDPSCs exposed to 3-Hz PW (**D–F**), and hDPSCs exposed to 300-Hz PW (**G–I**) were visualized. The scale bar is 10 µm.

**Figure 6:**
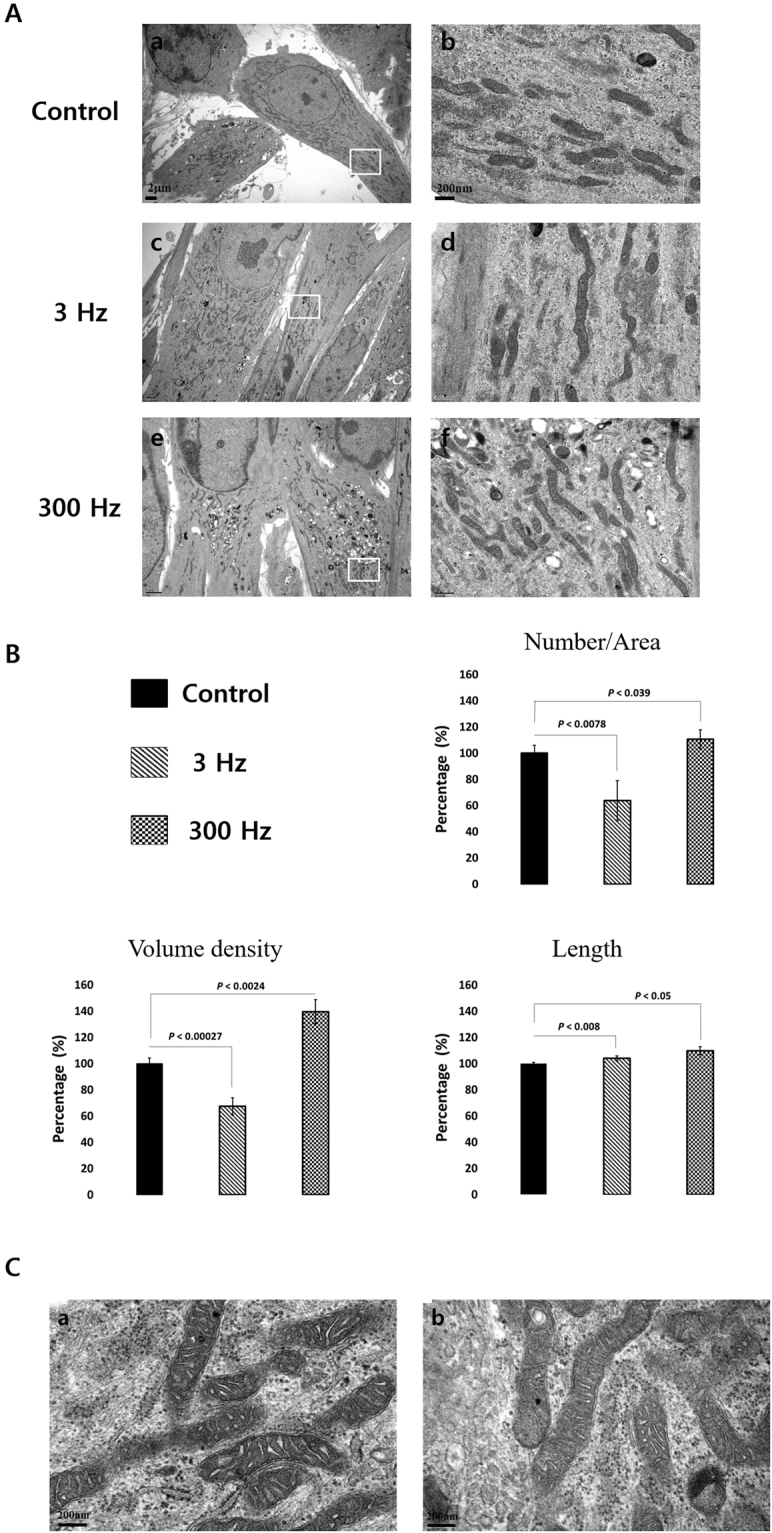
(**A**) Representative TEM images of mitochondria in hDPSCs after 7 days of incubation under osteogenic medium, 7 days after PBM treatment. hDPSCs (control, treated with 3-Hz PW-PBM, and treated with 300-Hz PW-PBM) were compared. The areas marked with white rectangles (a,c,e) were magnified (b,d,f). (**B**) Quantitative analyses of the number of mitochondria per area, mitochondrial volume density per cell, and mitochondrial length. (**C**) Magnified TEM image of mitochondria in control cells (a) and cells subjected to 300-Hz PW-PBM.

To determine whether PBM treatment triggers an alteration of the intracellular mitochondrial network in hDPSCs, we assessed the mitochondrial morphology of hDPSCs using MitoTracker® Red CMXRos (ThermoFisher Scientific, USA), which reflects mitochondrial shape. As shown in Fig. 5, our control sample contained small, rounded mitochondria. However, hDPSCs exposed to 3-Hz PBM contained elongated, tubular mitochondria. Interestingly, hDPSCs treated with 300-Hz PBM showed much more tubular mitochondrial morphology than hDPSCs exposed to 3-Hz PBM treatment. To confirm those results, we analyzed the alterations in mitochondrial shape using transmission electron microscopy (TEM). Figure 6A shows representative TEM images that reveal that hDPSCs subjected to 3-Hz PBM produced fewer mitochondria. Quantitative analysis showed that the number of mitochondria, normalized to the total area of each image, decreased significantly in hDPSCs exposed to 3-Hz PBM treatment, by 63.69 ± 15.18% (*P* < 0.007; Fig. 6B). In contrast, PBM treatment of 300 Hz promoted mitochondrial numbers, by 110.6 ± 17.28% (*P* < 0.039; Fig. 6B), compared to the control. In addition, mitochondrial volume density, defined as the volume occupied by mitochondria divided by the volume of the cytoplasm in percentage terms, decreased in hDPSCs subjected to 3-Hz PBM treatment, by 67.43 ± 6.53% (*P* < 0.00027; Fig. 6B). In contrast, the hDPSCs that received 300-Hz PBM treatment significantly promoted mitochondrial volume density, by 139.67 ± 9.09% (*P* < 0.0024; Fig. 6B), compared to the control. In addition, we found differences in the mitochondrial length of hDPSCs treated with 3- and 300-Hz PBM compared to controls. hDPSCs subjected to 300-Hz PBM treatment showed a mitochondrial length of 109.78 ± 3.17% (*P* < 0.05; Fig. 6B), compared to controls. Unlike the mitochondrial number and volume density, mitochondrial length increased, compared to controls, in the hDPSCs subjected to 3-Hz PBM treatment. However, we found no difference in the mitochondrial cristae structure between the controls and hDPSCs subjected to 300-Hz PBM treatment (Fig. 6C).

## Discussion

In PBM, pulsed wave light (PW) has been reported to be more effective in some biological systems than continuous wave light (CW)^40^. In particular, PW-PBM more effectively accelerated wound healing, reduced pain, and reduced ischemic stroke, apparently by enhancing ATP synthesis in the mitochondria^40,41^. It is reported that the effective pulse frequency range for PW-PBM is between 10 and 8,000 Hz^11,42^, with specific effective values for particular cell types. In the case of hDPSCs, no report had considered PW-PBM, even though CW-PBM showed great enhancement in hDPSC differentiation^21^. Therefore, our finding of effective pulse frequencies for hDPSC differentiation, especially in relation to mitochondrial activities, is the first such result.

In this study, we considered the relationship between the effectiveness of PBM and mitochondrial activity using DL, the luminescence emitted from living organisms exposed to external light. This lasts from a few seconds to minutes, and the initial intensity exceeds the spontaneous luminescence emitted without exposure to external light. The spontaneous luminescence of living organisms is called biophoton emission and occurs in the ultraviolet (UV) and NIR ranges^43^ with a low intensity of 10^−19^ to 10^−16^ W/cm^2^ ^44^. The endogenous production of excited molecules during oxidative metabolic reactions is known to be a source of biophoton emission^45^. Though both biophoton emission and DL exist concurrently, we cannot distinguish between the two signals because of the detection limit of our equipment. The detection limit is about 10^−10^ W/cm^2^, which is not good enough to detect ultraweak biophoton emissions without light exposure. Therefore, we analyzed our data as representing only DL.

Because of the strong correlation between the properties of DL and the states of biological systems, DL has been regarded as a powerful tool for medical investigations^27–29,34–39^. However information on the mechanisms for DL in animals is rather scarce, compared to that for plants^46^. Some hypotheses have been suggested. DL could be generated directly from autofluorescence emitters such as flavins^28^, from the solitons in hierarchically organized structures such as the cytoskeleton^47^, or from collective molecular interactions such as occur in mitochondrial membrane protein complexes^37^. Several papers have reported a close relationship between DL and mitochondria. Baran *et al*. showed that DL originates mainly from mitochondrial complex I, which they probed using complex I targeting agents^39^. They showed that complex I appeared to exhibit cooperative interactions among nicotinamide adenine dinucleotide, flavin mononucleotide, and their binding sites, emitting long-lasting DL with multiscale kinetics, as in plant chloroplasts. In our study, we analyzed DL signals one second after external light exposure, which should eliminate direct autofluorescence, whose lifetime is within a millisecond. Therefore, our DL signals are probably primarily from sequential photochemical reactions in some metastable-state species in mitochondria.

Our experimental data have some different points from those in previous studies. First, a complex III blocking agent (AMA) affected DL signals more than a complex I blocking agent (rotenone). We assume that this difference originated from the different wavelengths of light. Many studies used UV-VIS light, and their target molecules were flavins or nicotinamide adenine dinucleotide (NADH). Because we used NIR light in this study, the target molecules should be different. Cytochrome c and cytochrome c oxidase are known to absorb longer wavelength light, including NIR, which supports the role of complex III in NIR-induced DL^25^. Excited or metastable-state-cytochrome c could bind with complex III forwards or backwards depending on the environmental conditions, as observed in other DL systems. Second, the effects of antioxidants were much bigger than those observed in previous studies. In a previous study, the correlation between DL and ROS/lipofuscin was not particularly significant in UV-induced DL^39^. It is well known that PBM increases ROS production and that ROS have primary roles in the process of PBM^48^. The ROS might change the chemical environments of mitochondrial complex I or III or be directly bound to them, resulting in longer lasting DL signals following exposure to NIR light. The big reduction in DL seen with the addition of a ROS scavenger supports the relationship between ROS and PBM. Third, the DL we measured followed a single power law, which was confirmed by well fitted linear plots for *P*(*t*). In previous studies, DL generally presented multiscale kinetics that should be fitted using combinations of exponential components. Because we analyzed late period DL, we might have lost information about many quick, complex events. Nevertheless, our late period DL showed that the signal is from a few or a single event, which is meaningful information about PBM-induced changes in cell physiology.

One of the most important findings in this study is that pulsing PBM brings differential biological consequences according to the pulsing frequency. The DL lifetime was longer after PW-PBM irradiation, and the effects of rotenone, AMA, and NAC depended on the pulse frequency. Whereas cells treated with 30–300-Hz PW-PBM had higher and longer-lasting DL in all cases, cells treated with lower or higher frequency PW-PBM showed big changes when AMA and NAC were added. Though the exact molecular reactions to PW-PBM have not been clarified, we have found that certain ranges of pulse frequency have specific biological effects. 30–300-Hz PW-PBM seems to induce highly correlated photochemical reactions that are not easily affected by environmental redox changes. These periodic light stimuli might accelerate or retard some biological processes whose time scales are similar. Three biological processes can be suggested. One is a redox-linked proton pump of cytochrome c oxidase that is triggered by cytochrome c. Electrons from cytochrome c are transferred to the binuclear heme/copper (a_3_/Cu_B_) oxygen reduction site via a bimetallic Cu_A_ center at the membrane surface, which takes about a millisecond^49^. Another is the kinetics of ion channels, whose time scale is a few to a hundred milliseconds^50^. Ion concentrations in the mitochondrial membrane are a key factor controlling its functions. The third process is a synchronized whole-cell oscillation through the mitochondrial metabolism triggered by a local release of ROS^51^. It was reported that ROS efflux and antioxidant capacity determined the oscillation.

It should be noted that, among the tested frequencies of PW-PBM, 300-Hz PW had significant effects on hDPSC differentiation, as well as in chemical activations. The intracellular ROS analyzed via H2DCFDA was highest at 300-Hz PW-PBM. A similarly significant enhancement was observed in ALP activity, which is related to the early and middle stages of hDPSC differentiation into osteogenic lineages. The enhanced ALP activity and DL decreased when NAC was added, which implies that ROS played some role in 300-Hz PW-PBM-induced cellular responses. Our viability test showed that metabolic activities were not triggered by 300-Hz PW-PBM. Thus, this PBM stimulus might trigger cellular differentiation-related pathways rather than proliferation-related ones, which should be further studied.

Meanwhile, some studies report that activated complex I or III in mitochondrial electron transport chain (ETC) stimulates stem cells to differentiate^52,53^. Complex I or III in the electron transport chain is known to representatively produce superoxide anions. ROS resulting from those superoxide anions contribute to stem cell differentiation, which is different from stem cell type. Myoblasts increased more in complex I than in other complexes when progressing differentiation into muscle, and mouse embryonic stem cells enhanced the activity of complex III to cause other stem cells to differentiate. According to a previous study^25^, NIR light changes the mitochondrial metabolism for stem cell differentiation between complex III and complex IV via cytochrome c, resulting in the production of ROS and thereby activating transforming growth factor beta 1 to trigger stem cells to differentiate^21^. In the present study, complex III was activated more than complex I by 300-Hz PW-PBM, as shown by the results of the rotenone- and AMA-blocked decay probability, *P*(*t*), in Fig. 4. In addition, robust production of ROS was observed following 300-Hz PBM (Fig. 2B). This robust activation of complex III by 300-Hz PBM might be associated with the enhanced mitochondrial networks shown in Fig. 5 and the increased mitochondrial mass shown in Fig. 6. Taken together, we assume that 300-Hz PW-PBM activates ETC in complex III and produces more mitochondrial networks, which influence the differentiation of hDPSCs.

The mitochondrial activation caused by 300-Hz PW-PBM is supported by the changes in mitochondrial morphology and mass. TEM microscopy (Fig. 6a,b) demonstrated that 300-Hz PW-PBM induced a significant richness of mitochondrial mass by increasing the number and volume density of mitochondria in the hDPSCs. Though the underlying mechanisms for those changes have not been elucidated, relationships between some physiological observations and mitochondrial morphology and mass have been reported. For example, stem cells increased the production of fusion regulators (Mfn2 and Opa 1) and fission regulators (Fis 1 and Drp1) as differentiation progressed into osteogenesis, which resulted in an increase in mitochondrial mass and elongation^54^. This observation is consistent with our results that 300-Hz PW-PBM enhanced ALP activity, which accompanied increased mitochondrial mass and length. Another paper reported a negative relationship between mitochondrial elongation and mitochondrial ROS (mtROS)^55^. We demonstrated that the entire cytosolic ROS level was higher after 300-Hz PW-PBM (Fig. 2A). Though we did not include this result in this paper, we found that the level of mtROS was lower under the same conditions. This implies the differential activation of mitochondrial ETCs or the involvement of other mitochondrial redox regulators as a result of PW-PBM. Additionally, it was reported that PBM induced mitochondrial fission and fusion by regulating Ca^2+^ signaling, which has a close relationship with ROS^56^. Future, rigorously designed experiments should determine these cooperating phenomena. Because our microscopic observations were carried out 3 days after PW-PBM, it is difficult to state the exact relationships between our DL measurements and mitochondrial morphological changes. However, our DL screening data predict that 300-Hz PW would have the strongest effect on mitochondrial activation. In addition, we expect that 300-Hz PW-PBM produces some initiating signals that activate mitochondria and hDPSC differentiation. The activation of mitochondrial complex IV could play that initiating role by interacting with cytoplasmic ROS.

In summary, we found that 300-Hz PW-PBM dominantly prolonged the DL pattern and enhanced ALP activity, which consequently resulted in dentinogenic differentiation of hDPSCs. On the contrary, 3-Hz PW-PBM produced inverse effects on hDPSCs: it decreased the mitochondrial number and volume density, lowered the production of ROS, and produced little effect on DL. In PW-PBM therapy, pulsed waves were originally used only to reduce the thermal damage. However, our results reveal that the repetition frequency is an important factor in biological responses, as are wave frequency and power. These results can be used to enhance the efficiency of PBM treatment in many biological fields, including stem cell engineering.

## Materials and Methods

### Cell culture

hDPSCs were isolated from a tooth obtained following Institutional Review Board approval at Seoul National University Hospital (Seoul, South Korea; IRB number 05004). All three patients consented to the use of their teeth for research purposes, and no information about those patients is included in this article. This article does not contain information or images that could lead to the identification of study participants. All methods performed here were in accordance with the relevant regulations. The tooth was dissected aseptically and incubated with 4 ml of 0.25% trypsin-EDTA (Life Technologies) at 37 °C for 30 min. After neutralization with 4 ml of complete medium, solutions were pipetted vigorously to release cells and then passed through a cell strainer (70 mm, Corning). The resulting cells were cultured in complete medium supplemented with 100 mM ascorbic acid (Sigma). hDPSCs were cultured in a 37 °C incubator with 5% CO^2^ in complete medium composed of 10% fetal bovine serum (FBS), Dulbecco’s modified Eagle’s medium, and penicillin (100 U/ml)–streptomycin (100 μg/ml) (all from Gibco, Life Technologies) and supplemented with 100 mM ascorbic acid. For differentiation of hDPSCs into an osteogenic linage, 10 mM β-glycerophosphate, 0.05 mM L-ascorbic acid-2-phosphate, and 100 nM dexamethasone were added to the complete medium.

### PBM system and treatment

Unlike other light systems, our custom device was fabricated so that light from the LED propagated directly onto cells without a medium, removing the loss of light. It was composed of a diffuser, a light guide, a reflector, and an array of LEDs, as shown in Fig. 1. Seventeen LEDs whose wavelength was centered at 810 nm (PV810-3C6W-EDISAA, KAOS, Korea) were positioned at 1-cm intervals at one side the device. Light traveled through a disperser and a reflector, and the light intensity became uniform with a variance of less than 3.9% at the center area (marked with the white dotted square). The system was operated in a continuous wave (CW) mode or in a pulsed wave (PW) mode, with frequencies of 1, 3, 30, 300 Hz and 3 kHz. Constant DC voltage was supplied, and it was switched by an 8-bit micro controller device (UM_MC95FG308_V3.20_EN, Korea). The duty cycle was kept to 30% in all PW-PBM treatment modes. About 18.0 mA was applied to each LED, and the power density was 426 μW/cm^2^. Power density (irradiance, W/cm^2^) was measured by a power meter (PM-USB-100, Thorlabs, USA). The exposure time was determined so that the total energy was 38 mJ/cm^2^ for all experiments. The light dose was checked before each experiment. For long term exposure for hDPSC differentiation, PBM was conducted every day.

### Delayed luminescence spectroscopy

For DL spectroscopy, hDPSCs were first washed twice with DPBS to eliminate any influence of FBS. The light sensor (power range: 500 pW–0.5 mW, S130VC, Thorlabs, USA), with a resolution of 37 ms per datum, was placed on the PBM system in a CO_2_ incubator (MC-20A, Science & Technology Inc., Korea) in darkness for 30 min to remove natural luminescence. The light sensor’s area for detection was similar to the diameter of a 24-well plate.

To inhibit the mitochondrial respiratory chain components, we used 100 nM rotenone^57^ and 4 μM antimycin-A (AMA)^58^ to block the function of complex I and complex III, respectively, in the electron transport chain. hDPSCs were incubated with those solutions for 60 min, and then DL was measured. To test the role of ROS, the cells were treated with NAC for 30 min, and then DL was measured. All the measurements were conducted for 20 min, and all data were subtracted from the cell-free condition. The control (non-illuminated cells) DL data are not presented because their intensity level (10^−19^ to 10^−16^ W/cm^2^) was out of the range of the light sensor, representing spontaneous photon emissions^59^.

### Mathematical description of light-induced DL as a probability distribution

The relaxation of a non-equilibrium state into the equilibrium of a complex system can be approximated by a power law^34^. The DL temporal trend for a biological system can be modeled by a hyperbolic function, as given in ref.^35^:

1 $$I(t)=\frac{{I}_{0}}{{(1+t/{t}_{0})}^{m}}$$

where *I*(*t*) is the emitted intensity that can be obtained from the experimental data, *I*_0_ is the initial value of the emitted intensity, *t* is time, and *t*_0_ is an initial value.

DL intensity, *I*(*t*), is associated through the decay probability, *P*(*t*), with the degree of excitation, *n*(*t*), that attenuates at any time *t* by the expression *I*(*t*) = −*dn*(*t*)/*dt* ^34^. This can be evaluated for the value of a dimensionless function obtained from the experimental values of the intensity, *I*(*t*), at a time *t*, as follows^34^:

2 $$P(t)=(\frac{dn}{n}/\frac{dt}{t})=\frac{I(t)t}{n(t)}$$

where

3 $${\rm{n}}(t)={\int }_{t}^{\infty }I(t^{\prime} )dt^{\prime} \,n(t)=-(\frac{{I}_{0}{t}_{0}}{-m+1}){(1+\frac{t}{{t}_{0}})}^{-m+1}$$

Using the data from Fig. 2, *P*(*t*) and *n*(*t*) are obtained.

### Cell viability assay

hDPSCs were seeded in a 24-well plate with 2.5 × 10^4^ cells/well or in a 96-well plate with 1.0 × 10^4^ cells/well. 24 h after seeding, the hDPSCs were exposed to PW-PBM. Cell viability was assessed 10, 24, 48, and 72 hours after PBM treatment using the WST-1 assay (EZ-cytox, EZ-3000, Korea), as previously described^60^. For these experiments, a plate reader (Tecan, USA) measured the absorbance at 450 nm.

### Detection of intracellular ROS

To detect intracellular ROS in hDPSCs after PBM treatment, we used H2DCFDA (Molecular Probes). hDPSCs were seeded in a 4-well plate with 5 × 10^4^ cells/well. 24 h after seeding, the hDPSCs were exposed to PW-PBM. 20 minutes after starting the PBM treatment, the cells were incubated in media containing 10 μM H2DCFDA. After two washes with DPBS, the cells were incubated in complete medium for 30 minutes. Then the cells were trypsinized, and the fluorescence intensity was read via flow cytometry (BD Verse, Germany).

### Alkaline phosphatase assay

hDPSCs were seeded in a 96-well plate with 5.0 × 10^4^ cells/well in complete medium, and 24 h after seeding, they were treated with PW-PBM in osteogenic differentiation medium. 3, 7, and 14 days after PW-PBM treatment, ALP activity was assessed using the SensoLyte® pNPP Alkaline Phosphatase Assay Kit (AnaSpec, USA) following the manufacturer’s instructions.

### Electron transmission microscopy

To optimize mitochondrial structural preservation and membrane contrast, cells were fixed with 2% paraformaldehyde and 2.5% glutaraldehyde (Ted Pella, Redding, CA, USA) in 0.15 M sodium cacodylate (pH 7.4) at 37 °C and placed in pre-cooled fixative on ice for 1 h. The cells were post-fixed with 1% osmium tetroxide, 0.8% potassium ferrocyanide, and 3 mM calcium chloride in 0.1 M sodium cacodylate (pH 7.4) for 1 h, washed with ice-cold distilled water, post-stained with 2% uranyl acetate at 4 °C, dehydrated using graded ethanol, and embedded in Durcupan resin (Fluka, St. Louis, MO, USA). Ultrathin (70 nm) sections were post-stained with uranyl acetate and lead salts and observed using a JEOL 1200FX (JEOL, Japan) at 80 kV. Images were digitized at 1,800 dpi using a Nikon Cool scan system (Nikon Instruments Inc., USA), giving an image pixel array of 4,033 × 6,010 and a pixel resolution of 1.77 nm. Mitochondrial lengths were measured with ImageJ.

### Mitochondrial bioenergetics

The mitochondrial mass was measured with MitoTracker® Red CMXRos (ThermoFisher Scientific), which binds to cardiolipin in the mitochondrial inner membrane. hDPSCs were seeded onto coverslips in a 4-well plate with 1.0 × 10^4^ cells/well. 24 h after seeding, the hDPSCs were exposed to PW-PBM. 3 days after PBM treatment, the cells were incubated in serum-free medium containing 50 nM MitoTracker for 30 minutes. After washing with Dulbecco’s phosphate-buffered saline (DPBS), cells were fixed with 4% PFA and observed with confocal microscopy (LSM-SP8X, Calzeiss, Germany).

To confirm the fluorescence data, the TEM images were analyzed. The mitochondrial volume density, defined as the volume occupied by mitochondria divided by the volume occupied by the cytoplasm, was estimated using stereology as follows. A 112 × 112 square grid (chosen for ease of use) was overlaid on each image in Photoshop (Adobe Systems Inc., USA), and the mitochondria and cytoplasm lying under the intercepts were counted. The relative volume of the mitochondria was expressed as the ratio of intercepts coinciding with this organelle relative to the intercepts coinciding with the cytoplasm.

### Statistical analysis

Data were analyzed using Microsoft Excel. Means and standard deviations were calculated. Because many measurement variables in biology fit the normal distribution, we performed an unpaired two-sided student’s t test for all data. All the experiments were repeated more than four times, and the standard deviations were plotted in the graph. We considered results statistically significant when *P* < 0.01 and *P* < 0.05.
